# Protein deubiquitinase USP7 is required for osteogenic differentiation of human adipose-derived stem cells

**DOI:** 10.1186/s13287-017-0637-8

**Published:** 2017-08-14

**Authors:** Yiman Tang, Longwei Lv, Wenyue Li, Xiao Zhang, Yong Jiang, Wenshu Ge, Yongsheng Zhou

**Affiliations:** 1 Department of Prosthodontics, Peking University School and Hospital of Stomatology, 22 Zhongguancun Avenue South, Haidian District, Beijing, 100081 People’s Republic of China; 2 Department of General Dentistry II, Peking University School and Hospital of Stomatology, 22 Zhongguancun Avenue South, Haidian District, Beijing, 100081 People’s Republic of China; 3 National Engineering Laboratory for Digital and Material Technology of Stomatology, Beijing Key Laboratory of Digital Stomatology, Beijing, 100081 China

**Keywords:** Ubiquitin specific protease 7, Osteogenic differentiation, Human adipose-derived stem cells, Deubiquitinase, Bone engineering

## Abstract

**Background:**

Human adipose-derived stem cells (hASCs) are multipotent progenitor cells with self-renewal capabilities and multilineage differentiation potential, including osteogenesis. Although protein deubiquitinases have been linked to stem cell fate determination, whether protein deubiquitination contributes to lineage commitment during osteogenic differentiation of hASCs remains to be investigated. The objective of this study was to evaluate the effects of the ubiquitin specific protease 7 (USP7) on osteogenic differentiation of hASCs.

**Methods:**

An osteocalcin promoter driven luciferase reporter system was established to initially discover the potential association between USP7 and hASC osteogenesis. To further characterize the function of USP7 in osteogenic differentiation of hASCs, a combination of in vitro and in vivo experiments were carried out through genetic depletion or overexpression of USP7 using a lentiviral strategy. Moreover, HBX 41,108, a cyanoindenopyrazine-derived deubiquitinase inhibitor of USP7, was utilized at different doses to further examine whether USP7 regulated osteogenic differentiation of hASCs through its enzymatic activity.

**Results:**

We demonstrated that USP7 depletion was associated with remarkable downregulation of the reporter gene activity. Genetic depletion of USP7 by lentiviral RNAi markedly suppressed hASC osteogenesis both in vitro and in vivo, while overexpression of USP7 enhanced the osteogenic differentiation of hASCs. Notably, chemical blockade via the small molecular inhibitor HBX 41,108 could efficiently mimic the effects of USP7 genetic depletion in a dose-dependent manner.

**Conclusions:**

Taken together, our study revealed that protein deubiquitinase USP7 is an essential player in osteogenic differentiation of hASCs through its catalytic activity, and supported the pursuit of USP7 as a potential target for modulation of hASC-based stem cell therapy and bone tissue engineering.

**Electronic supplementary material:**

The online version of this article (doi:10.1186/s13287-017-0637-8) contains supplementary material, which is available to authorized users.

## Background

Human adipose-derived stem cells (hASCs), adult mesenchymal stem cells (MSCs) with multiple differentiation potentials and self-renewal capabilities [1, 2], have become highly attractive sources in bone tissue engineering owing to the advantages of good accessibility, rapid proliferation, and little donor site suffering [3, 4]. How to effectively promote osteogenic differentiation of hASCs has always been a core issue in the bone regeneration field. Osteogenic differentiation of hASCs is believed to be a complex process tightly regulated by multiple layers of regulation including transcriptional and post-transcriptional levels. Recently, post-transcriptional protein modification including phosphorylation, acetylation, and methylation [5, 6], which plays pivotal roles in self-renewal and fate determination of stem cells, has come to the forefront of research on lineage commitment of hASCs [7, 8]. Our previous studies reported that histone demethylase retinoblastoma binding protein 2 (RBP2) and lysine-specific demethylase 1 (LSD1) inhibited osteogenic differentiation of hASCs through repression of osteogenesis-related gene expression via their catalytic activities [9, 10].

Similar to methylation, protein ubiquitination is also a reversible modification and has been implicated in stem cell differentiation and organ development or homeostasis [11]. In humans, a large family of deubiquitinases (DUBs) act to oppose protein ubiquitination through hydrolyzing the ubiquitin linkages, thereby controlling the function or abundance of targeted proteins and influencing physiological or pathological processes [12, 13]. Among these DUBs, ubiquitin specific protease 7 (USP7), also known as herpes virus-associated ubiquitin-specific protease (HAUSP), is one of the most extensively studied DUBs. Accumulating studies have shown that USP7 is able to target different proteins to regulate a series of biological processes including the immune response [14], virus replication and infection [15], mitosis [16], DNA replication [17], and DNA damage repair [18]. Meanwhile, the abnormal expression or functional dysregulation of USP7 is associated with pathological processes such as neurodegenerative diseases [19], inflammation [20], and tumors [21, 22]. Currently, USP7 has been reported to stabilize the repressor element 1-silencing transcription factor (REST) and is involved in the maintenance of neural progenitor cells [23]. However, the roles of USP7 on fate determination or orientated differentiation of MSCs remain to be investigated.

In this study, we investigated the previously unrecognized biological and functional roles of protein deubiquitinase USP7 in osteogenic differentiation of hASCs, both in vitro and in vivo, and explored whether USP7 regulated osteogenesis through its catalytic activity. We believe our findings will facilitate the development of a drug or small molecule to act on USP7 to modulate osteogenic differentiation of MSCs.

## Methods

### Cell culture and reagents

Primary hASCs and human bone marrow-derived mesenchymal stem cells (hBMMSCs) were purchased from ScienCell Company (San Diego, CA, USA). Stem cells used in the cell-based experiments were collected from three donors, and all the in vitro experiments were repeated in triplicate. All materials were purchased from Sigma-Aldrich (St. Louis, MO, USA) unless specially mentioned. Cells were cultured in proliferation media (PM) consisting of Dulbecco’s modified Eagle’s medium (DMEM; Gibco, Grand Island, NY, USA), 10% fetal bovine serum (ScienCell), and 100 IU/ml penicillin/streptomycin (Gibco). Osteogenic differentiation of cells was induced when cells grew to 80–90% confluence with osteogenic media (OM) containing standard PM supplemented with 100 nM dexamethasone, 0.2 mM ascorbic acid, and 10 mM β-glycerophosphate. Adipogenic differentiation of cells was induced when cells grew to 100% confluence with adipogenic media (AM) containing standard PM supplemented with 10 μM insulin, 1 μM dexamethasone, 0.5 mM 3-isobutyl-1-methylxanthine, and 200 μM indomethacin. HBX 41,108 was purchased from Tocris (MN, USA).

### Reporter vector construction and luciferase reporter assay

The genomic DNA harboring promoter regions of osteocalcin (*OC*_pro_) and DNA sequences encoding luciferase genes were amplified and cloned into the pLVX-pTRE-puro vector. Then, the generated *OC*_pro_-Luc-Puro construct together with three assistant vectors were transiently transfected into HEK293T cells followed by viral supernatant collection, filtration, and concentration. Next, the hASCs stably expressing the luciferase reporter gene driven by osteocalcin promoter were created with the infection of the lentivirus carrying *OC*_pro_-Luc-Puro cassette. For luciferase assays, the *OC*_pro_-Luc-hASCs were then cultured in PM or OM for 7 and 14 days. Briefly, the cells were washed with phosphate-buffered saline (PBS) two times, and incubated with 100 μL of cell lysate (PLB) per well for 15 min at room temperature (RT). LAR II solution was prepared by dissolving the luciferase substrate freeze-dried powder into 10 mL luciferase assay buffer and stored at –70 °C; 20 μL cell lysate and 50 μL LAR II were added into a 96-well fluorometric plate, and luciferase activities were measured by a luciferase reporter system (Progema).

### Lentivirus infection and establishment of stably expressing transductants

All recombinant lentiviruses were obtained from GenePharma Company (Shanghai, China). The packaged lentiviruses used contained lentiviruses targeting USP7 (shUSP7-1 and shUSP7-2), the scrambled control (shNC), full-length FLAG tagged USP7/wild-type (WT), and the scrambled control (Vector). According to the manufacturer’s procedure, hASCs and hBMMSCs were infected with the viral supernatants at a multiplicity of infection (MOI) of 100 associated with the presence of polybrene (5 μg/ml). Puromycin at 1 mg/ml was used for 4–7 days to select infected cells. The shRNA target sequences were as follows: shNC, TTCTCCGAACGTGTCACGT; shUSP7-1, TGTATCTATTGACTGCCCTTT; and shUSP7-2, CGTGGTGTCAAGGTGTACTAA.

### Alkaline phosphatase (ALP) staining and quantification

The cells cultured for 7 days with PM or OM were evaluated for ALP staining and quantification as previously described [24]. ALP staining was conducted using an NBT/BCIP staining kit (CoWin Biotech, Beijng, China). For quantification of ALP activity, the cells were lysed by 1% Triton X-100, sonicated on ice, and then centrifuged at 4 °C for 30 min at 12,000 g. Total protein contents were determined using the bicinchoninic acid method with the Pierce protein assay kit (Thermo Scientific, Rockford, IL, USA). ALP activity in aliquots of the same samples were measured using an ALP assay kit (Nanjing Jiancheng Bioengineering Institute, Nanjing, China). ALP levels relative to the control group were calculated after being normalized by the total protein content.

### Alizarin red S (AZR) staining and quantification

The cells cultured for 14 days with PM or OM were assayed for matrix mineralization. Briefly, the cells were first washed three times with PBS, fixed in 95% ethanol for 30 min, and then incubated with 0.1% Alizarin red S pH 4.2 for 1 h at RT. For quantification of mineralization, the calcium-bound AZR was dissolved in 100 mM cetylpyridinium chloride for 1 h. The absorbance of the released AZR was measured at 562 nm.

### Oil red O staining and quantification

After 14 days of adipogenic differentiation, the cells were washed with PBS three times and fixed in 10% formalin for 1 h. The cells were then rinsed with 60% isopropanol and stained with filtered 0.3% Oil red O solution at RT. After staining, the cells were washed with distilled water to remove unbound dye, visualized under a microscope (Olympus, Tokyo, Japan), and photographed. For quantitative assessment, the Oil red O was dissolved in 100% isopropanol and the absorbance was measured at 520 nm.

### Immunofluorescence staining

The hASCs were seeded on the sterile glass coverslips loaded on 12-well plates. After 14 days of culture with PM or OM, the samples were rinsed three times with PBS, fixed in 4% paraformaldehyde for 10 min at RT, and permeabilized with 0.1% Triton X-100 for 10 min. Then, the samples were incubated with 1:200 anti-osteocalcin antibodies (OC; Abcam, Cambridge, UK) at 4 °C overnight, followed by incubated with 1:1000 anti-rabbit secondary antibodies (Cell Signaling Technology, Danvers, MA, USA) for 1 h at RT. After staining the nuclei with DAPI, the coverslips were mounted on glass slides and viewed under a confocal Zeiss Axiovert 650 microscope using the laser with wavelengths of 488 nm (green, OC) and 405 nm (blue, DAPI).

### Quantitative reverse-transcription PCR (qRT-PCR)

Total cellular RNA was isolated using Trizol reagent (Invitrogen, Carlsbad, CA, USA), and subsequently reverse-transcribed into first-strand cDNA with a Reverse Transcription System (Takara, Kusatsu, Shiga, Japan). qRT-PCR was performed using a Power SYBR Green PCR Master Mix (Roche, Mannheim, Germany) and a 7500 Real-Time PCR Detection System (Applied Biosystems, Foster City, CA, USA). The internal standard for mRNA was GAPDH. The primer sequences were as follows: *GAPDH*, (forward) 5’-GGAGCGAGATCCCTCCAAAAT-3’ and (reverse) 5’-GGCTGTTGTCATACTTCTCATGG-3’; *USP7*, (forward) 5’-CCCTCCGTGTTTTGTGCGA-3’ and (reverse) 5’-AGACCATGACGTGGAATCAGA-3’; *RUNX2*, (forward) 5’-CCGCCTCAGTGATTTAGGGC-3’ and (reverse) 5’-GGGTCTGTAATCTGACTCTGTCC-3’; *ALP*, (forward) 5’-ATGGGATGGGTGTCTCCACA-3’ and (reverse) 5’-CCACGAAGGGGAACTTGTC-3’; *OC*, (forward) 5’-AGCAAAGGTGCAGCCTTTGT-3’ and (reverse) 5’-GCGCCTGGGTCTCTTCACT-3’; *OSX*, (forward) 5’-CCTCCTCAGCTCACCTTCTC-3’ and (reverse) 5’- GTTGGGAGCCCAAATAGAAA-3’. The cycle threshold (Ct) values were applied to calculate the fold changes by the 2^△△Ct^ method.

### Western blotting

The cells cultured for 14 days with PM or OM were assayed for Western blotting as previously described [25]. Briefly, the cells were treated in RIPA buffer for cell lysis, followed by sonicated and centrifuged to obtain suspensions. SDS-PAGE was conducted to separate proteins of different molecular weight, and then proteins were transferred to polyvinylidene difluoride membranes. Thereafter, the membranes were incubated in primary antibodies against RUNX2 (Cell Signaling Technology, Danvers, MA, USA), OSX (Abcam), USP7 (Bethyl, Montgomery, Alabama, USA), and GAPDH (Abcam) at 4 °C overnight, and then corresponding secondary antibodies for 1 h at RT. An ECL kit (CWBIO, Beijing, China) was applied to visualize the immunoreactive protein bands.

### Heterotopic bone formation in vivo

The in vivo study was approved by the Institutional Animal Care and Use Committee of the Peking University Health Science Center (LA2014233). The hASCs transfected with lentivirus (shNC, shUSP7-1, and shUSP7-2) were induced in OM for 7 days before the in vivo study. After being resuspended by trypsin, 1 × 10^6^ cells were incubated with 40 mg Synthograft™, a beta-tricalcium phosphate (β-TCP) synthetic bone graft (Bicon, Boston, MA, USA) [26], for 1 h at 37 °C, followed by centrifugation at 150 g for 5 min. The scaffolds were then transplanted subcutaneously into the dorsa of 5-week-old BALB/c homozygous nude (nu/nu) mice. Two transplantation sites were prepared in each mouse, providing randomly for the transplantation of three groups of cells: hASCs/shNC, hASCs/shUSP7-1, and hASCs/shUSP7-2 (*n* = 8 per group). Eight weeks after surgery, specimens were harvested and fixed in 4% paraformaldehyde for further experiments.

### Micro-computed tomography (micro-CT) analysis of xenograft mice

To assess the mass and shape of the new bone among the three groups, the specimens were scanned with an Inveon MM system (Siemens, Munich, Germany) after fixation as previously described [27]. The scanning conditions were an X-ray voltage of 80 kV, current of 500 μA, and exposure time of 1500 ms for each of the 360 rotational steps. For quantification of the images, bone volume/total volume (BV/TV) [28] was calculated using an Inveon Research Workplace (Siemens).

### Hematoxylin and eosin (H&E) staining, Masson’s trichrome, and immunohistochemical analysis

The specimens were fixed in 4% paraformaldehyde for 24 h and decalcified in 10% EDTA (pH 7.4) for 14 days, followed by dehydration and infiltration in paraffin. Sections (4-μm thickness) were cut and stained with H&E and Masson’s trichrome. Immunohistochemical analysis for osteocalcin (OC; Abcam) was also performed to evaluate osteogenesis. All the histological slices were visualized under a light microscope (Olympus, Tokyo, Japan). For quantification of bone-like tissue, two images of each sample (16 images for each group) were taken randomly. The percentage of new bone formation area—(bone area/total tissue area) × 100%—or mean density—integrated optical density of positive staining/cell containing tissue area—of immunohistochemical staining were measured by Image-Pro Plus software (Media Cybernetics, Rockville, MD, USA). Box plots were used to exhibit the quantitative results.

### Cell viability and apoptosis assays

The cell viability was evaluated with a Cell Counting Kit-8 (CCK8; Dojindo, Kumamoto, Japan). Cells were seeded at 5 × 10^3^ cells per well in 48-well plates and cultured in PM or OM with indicated treatment. At each time point, the supernatant of each group was removed, and cells were incubated with DMEM medium containing CCK-8 for 2 h at 37 °C. Optical density (OD) was measured at 450 nm using a microplate reader (ELX808, BioTek). Cell apoptosis was examined with Annexin V-FITC Apoptosis Detection Kit (Dojindo, Kumamoto, Japan) according to the manufacture’s protocol. Analysis was performed with a FACScan flow cytometer (Beckman Coulter, CA, USA) using the EXPO32 ADC software.

### Statistical analysis

Statistical results were analyzed by SPSS 20.0 (IBM, Armonk, NY, USA) software. Data from triplicate in vitro experiments were presented as mean ± standard deviation (SD). The independent two-tailed Student’s *t* test was applied for comparisons between two groups. The value of *P* < 0.05 was considered statistically significant.

## Results

### Protein deubiquitinase USP7 is potentially associated with hASC osteogenesis

**Fig. 1:**
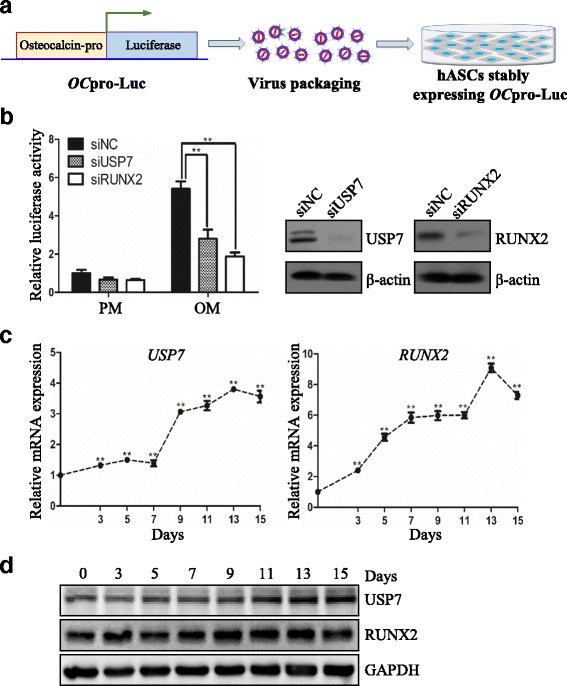
Protein deubiquitinase USP7 is potentially associated with hASC osteogenesis. **a** Schematic diagram of *OC*_pro_-Luc-hASC construction. **b** *OC*_pro_-Luc-hASCs were transfected with siRNA against USP7, with the scrambled siRNA as negative control and siRNA against RUNX2 as positive control. *Left panel*: Luciferase reporter assay with cellular extracts from *OC*_pro_-Luc-hASCs cultured in proliferation or osteogenic media for 7 days. *Middle and right panel*: Validation of USP7 and RUNX2 knockdown effect by Western blotting, respectively. **c** Relative mRNA expression of *USP7* and *RUNX2* measured by qRT-PCR during osteogenic differentiation of hASCs (normalized by *GAPDH*; relative to day 0 groups). **d** Western blotting of protein expression of USP7, RUNX2, and the internal control GAPDH during osteogenic differentiation of hASCs. Results are presented as the mean ± SD, *n* = 3. *P < 0.05, **P < 0.01. *hASC* human adipose-derived stem cell, *Luc* luciferase, *NC* negative control, *OC* osteocalcin, *OM* osteogenic media, *PM* proliferation media, *RUNX2* runt-related transcription factor 2, *USP7* ubiquitin specific protease 7

In order to identify potential deubiquitinases involved in osteogenesis, we established a luciferase reporter system driven by the promotor of osteocalcin (*OC*_pro_) (*OC*_pro_-Luc) and stably integrated the *OC*_pro_-Luc cassette into hASCs (Fig. 1a). Next, different siRNAs targeting multiple deubiquitinases were transfected into the *OC*_pro_-Luc-hASCs, with the scrambled siRNA as negative control and siRNA against RUNX2 as positive control. Cells cultured in proliferation media (PM) or osteogenic media (OM) were collected and luciferase activities were quantified. The results revealed that, similar to RUNX2 knockdown, USP7 depletion significantly downregulated the luciferase activity of *OC*_pro_-Luc-hASCs compared with control siRNA transfected cells (Fig. 1b). The knockdown effect of USP7 and RUNX2 was determined by Western blotting (Fig. 1b).

In support of this deduction, qRT-PCR analysis revealed that the endogenous expression level of *USP7* in hASCs was gradually elevated within 15 days after osteogenic induction. A similar pace was observed for the osteogenic marker *RUNX2*, the master transcription factor of osteogenic differentiation (Fig. 1c). Moreover, the dynamic pattern of protein abundance of these genes was also consistent, as indicated by Western blotting analysis (Fig. 1d), pointing to a role of USP7 in osteogenic differentiation.

### Knockdown of USP7 inhibits osteogenic differentiation of hASCs in vitro

**Fig. 2:**
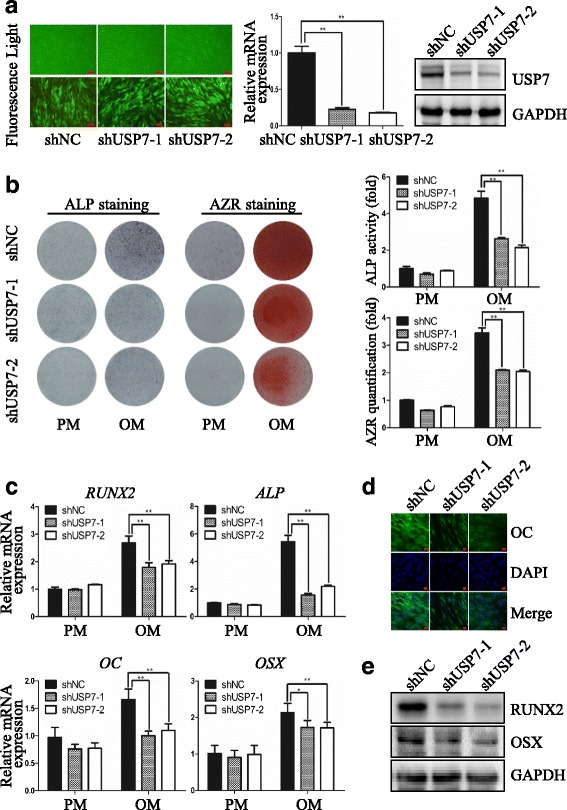
Knockdown of USP7 inhibits osteogenic differentiation of hASCs in vitro. **a** *Left panel*: Microscopic images of GFP-positive hASCs under ordinary and fluorescent light. *Scale bars* =100 μm. *Middle and right panel*: Validation of USP7 knockdown effect by qRT-PCR and Western blotting, respectively. **b** *Left panel*: Images of ALP staining on day 7, and AZR staining on day 14 in shNC, shUSP7-1, and shUSP7-2 groups treated with proliferation or osteogenic media. *Right panel*: Histograms show ALP activity and quantification of AZR staining by spectrophotometry. **c** Relative mRNA expression of *RUNX2*, *ALP*, *OC*, and *OSX* measured by qRT-PCR on day 14 of osteogenic induction. *GAPDH* was used for normalization. **d** Confocal microscopy of OC with DAPI counterstaining in shNC, shUSP7-1, and shUSP7-2 groups on day 14 of osteogenic induction. *Scale bars* = 50 μm. **e** Western blotting of the protein expression of RUNX2, OSX, and the internal control GAPDH on day 14 of osteogenic induction with antibodies as indicated. Results are presented as the mean ± SD, *n* = 3. *P < 0.05, **P < 0.01. *ALP* alkaline phosphatase, *AZR* alizarin red S, *GFP* green fluorescent protein, *hASC* human adipose-derived stem cell, *NC* negative control, *OC* osteocalcin, *OM* osteogenic media, *OSX* osterix, *PM* proliferation media, *RUNX2* runt-related transcription factor 2, *USP7* ubiquitin specific protease 7

To validate the potential role of USP7 in osteogenic differentiation of hASCs, we then generated hASCs stably expressing USP7 shRNAs. In this regard, two shRNAs targeting distinct regions of USP7 were designed to limit possible off-target effects. After osteogenic stimulation for 7 days, ALP activity in USP7-deficient cells was significantly decreased compared to control cells (Fig. 2b). Meanwhile, extracellular matrix mineralization, manifested by Alizarin red S staining and quantification on day 14, was also markedly reduced in USP7-deficient cells (Fig. 2b). The knockdown efficiency was analyzed by qRT-PCR and Western blotting, both of which showed that 70% of endogenous *USP7* was eliminated compared with the control treatment (Fig. 2a). These results indicated that USP7 is essential for osteogenic differentiation of hASCs.

Next, we investigated whether impairment of osteogenic differentiation resulting from USP7 deficiency is associated with altered expression of osteogenic genes. To this end, control hASCs or hASCs with USP7 knockdown by lentivirus shRNA were cultured in PM or OM. Total RNAs were collected and analyzed by qRT-PCR. As shown in Fig. 2c, the expression level of osteogenic markers such as *ALP*, *RUNX2*, *OC*, and *OSX* was upregulated after osteogenic induction, while this effect was significantly compromised on USP7 knockdown. Consistently, Western blotting analysis indicated that the protein level of RUNX2 and OSX was reduced in USP7-deficient cells (Fig. 2e), while immunofluorescence staining demonstrated that the protein expression level of OC was also downregulated (Fig. 2d). These observations indicated that inhibition of osteogenic differentiation in USP7 depletion cells is associated with decreased expression of these osteogenic genes.

Taken together, these results indicated that USP7 knockdown inhibits osteogenic differentiation of hASCs in vitro.

### Overexpression of USP7 promotes osteogenic differentiation of hASCs in vitro

**Fig. 3:**
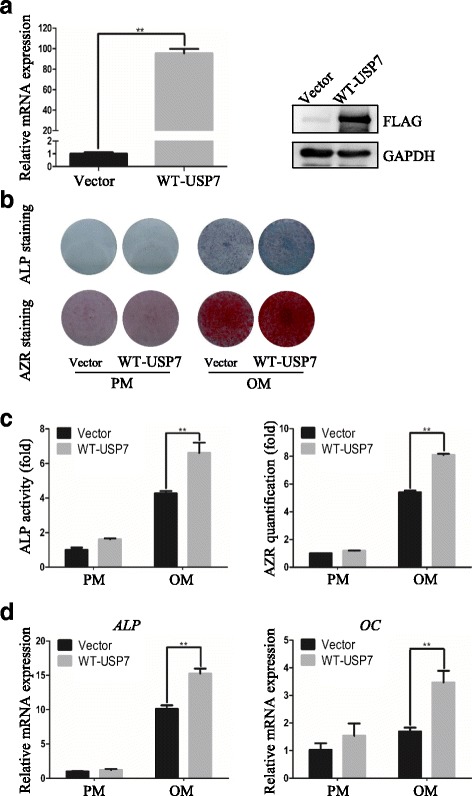
Overexpression of USP7 promotes osteogenic differentiation of hASCs in vitro*.* **a** Validation of USP7 overexpression effect by qRT-PCR and Western blotting, respectively. **b** Images of ALP staining on day 7, and AZR staining on day 14 in control or USP7 overexpression cells treated with proliferation or osteogenic media. **c** Histograms show ALP activity and quantification of AZR staining by spectrophotometry. **d** Relative mRNA expression of *ALP* and *OC* measured by qRT-PCR on day 14 of osteogenic induction. GAPDH was used for normalization. Results are presented as the mean ± SD, *n* = 3. *P < 0.05, **P < 0.01. *ALP* alkaline phosphatase, *AZR* alizarin red S, *hASC* human adipose-derived stem cell, *OC* osteocalcin, *OM* osteogenic media, *PM* proliferation media

To further confirm the function of USP7 in osteogenesis, we established USP7 overexpression cells with lentivirus carrying FLAG tagged USP7/wild-type (WT). According to the results, qRT-PCR analysis of USP7 expression confirmed a nearly 100-fold increase in the USP7 overexpression group compared with the control group, and Western blotting analysis displayed consistent protein levels (Fig. 3a, Additional file 1: Figure S1). After osteogenic differentiation for 7 days, ALP activity was significantly increased in USP7 overexpression cells (Fig. 3b and c). The extracellular matrix mineralization of hASCs, as measured by Alizarin red S staining and quantification on day 14, was also increased (Fig. 3b and c). In addition, qRT-PCR analysis revealed that USP7 overexpression significantly upregulated the *ALP* and *OC* mRNA levels (Fig. 3d). Taken together, these results indicated that USP7 promotes osteogenic differentiation of hASCs in vitro.

### USP7 inhibitor HBX 41,108 compromises osteogenic differentiation of hASCs

**Fig. 4:**
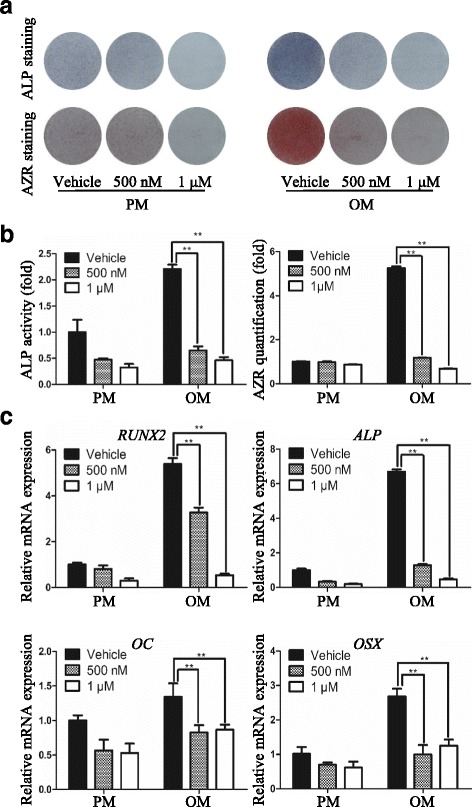
USP7 inhibitor HBX 41,108 compromises osteogenic differentiation of hASCs. **a** Images of ALP staining on day 7, and AZR staining on day 14 of hASCs treated with proliferation or osteogenic media in the presence of vehicle or HBX 41,108. **b** Histograms show ALP activity and quantification of AZR staining by spectrophotometry. **c** qRT-PCR analysis of osteogenesis associated genes *RUNX2*, *ALP*, *OC*, and *OSX* expression in the presence of vehicle or HBX 41,108 on day 14 after osteoinduction. *GAPDH* was used for normalization. Results are presented as the mean ± SD, *n* = 3. *P < 0.05, **P < 0.01. *ALP* alkaline phosphatase, *AZR* alizarin red S, *hASC* human adipose-derived stem cell, *OC* osteocalcin, *OM* osteogenic media, *OSX* osterix, *PM* proliferation media, *RUNX2* runt-related transcription factor 2

The phenoptype that USP7 depletion resulted in diminution of osteogenic differentiation of hASCs prompted us to further test whether this effect is dependent on its deubiquitinase activity. Recently, small molecular drugs as inhibitors of epigenetic regulating enzymes have attracted increasing attention due to their safety and convenience compared with virus infection [29]. Therefore, we treated hASCs with HBX 41,108, a cyanoindenopyrazine-derived deubiquitinase inhibitor which inhibits catalytic activity of USP7 [30]. The activity of ALP, determined by ALP staining (Fig. 4a) and quantification (Fig. 4b), was reduced in hASCs exposed to HBX 41,108. Similar results were obtained when the extracellular matrix mineralization effect was assessed by Alizarin red S staining (Fig. 4a) and quantification (Fig. 4b). In addition, qRT-PCR results demonstrated that HBX 41,108 treatment led to a dramatic decrease of *ALP*, *RUNX2*, *OC*, and *OSX* expression in a dose– dependent manner (Fig. 4c). Furthermore, we investigated the effects of HBX 41,108 on the proliferation and apoptosis of hASCs. Results revealed that HBX 41,108 at concentrations of less than or equal to 1 μM had no obvious cellular toxicity or cellular growth retardation, as shown in Additional file 2: Figure S2. Collectively, these data indicated that enzymatic inhibition of USP7 phenocopies the effects of USP7 knockdown, confirming the inhibitory effect of this small molecule and supporting that USP7 functions as an essential factor in osteogenic differentiation of hASCs through its catalytic activity.

### Knockdown of USP7 or USP7 inhibitor HBX 41,108 application suppresses osteogenic differentiation of hBMMSCs

**Fig. 5:**
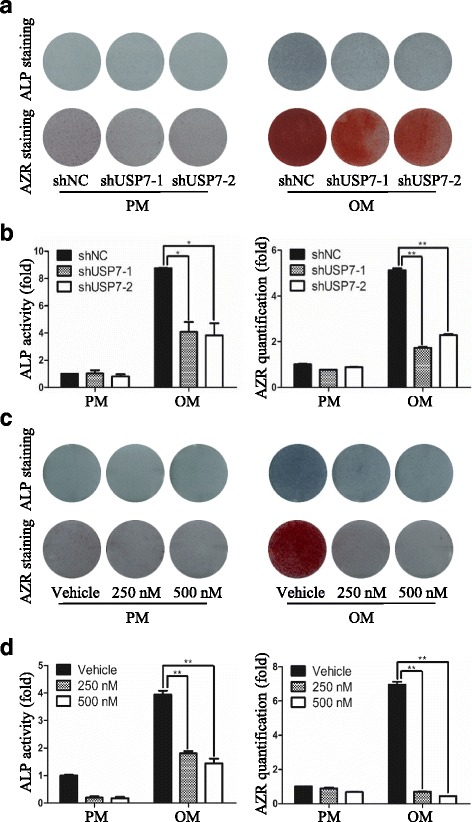
Knockdown of USP7 or USP7 inhibitor HBX 41,108 application suppresses osteogenic differentiation of hBMMSCs. **a** Images of ALP staining on day 7, and AZR staining on day 14 of hBMMSCs expressing shNC, shUSP7-1, or shUSP7-2 after osteoinduction. **b** ALP activity on day 7 and quantification of AZR staining on day 14 of hBMMSCs expressing shNC, shUSP7-1, or shUSP7-2 after osteoinduction. **c** Images of ALP staining on day 7, and AZR staining on day 14 of hBMMSCs cultured in the absence or presence of HBX 41,108 after osteoinduction. **d** ALP activity on day 7 and quantification of AZR staining on day 14 of hBMMSCs cultured in the absence or presence of HBX 41,108 after osteoinduction. Results are presented as the mean ± SD, *n* = 3. *P < 0.05, **P < 0.01. *ALP* alkaline phosphatase, *AZR* alizarin red S, *hBMMSC* human bone marrow-derived mesenchymal stem cell, *NC* negative control, *OM* osteogenic media, *PM* proliferation media, *USP7* ubiquitin specific protease 7

To further corroborate the finding that USP7 is an essential player in osteogenic differentiation, we examined the effects of USP7 on the osteogenic commitment of another type of mesenchymal stem cell, human bone marrow-derived mesenchymal stem cells (hBMMSCs) [31]. Knockdown of USP7 significantly reduced the osteogenic differentiation manifested by both ALP and Alizarin red S staining and quantification (Fig. 5a and b). Consistently, the ALP activity and extracellular matrix mineralization effect (Fig. 5c and d) were both decreased in HBX 41,108-treated hBMMSCs in a dose-dependent manner upon osteogenic differentiation. In summary, these data indicated that, analogous to its effect on hASCs, USP7 deficiency inhibits osteogenic differentiation of hBMMSCs.

### Knockdown of USP7 impairs bone formation in vivo

**Fig. 6:**
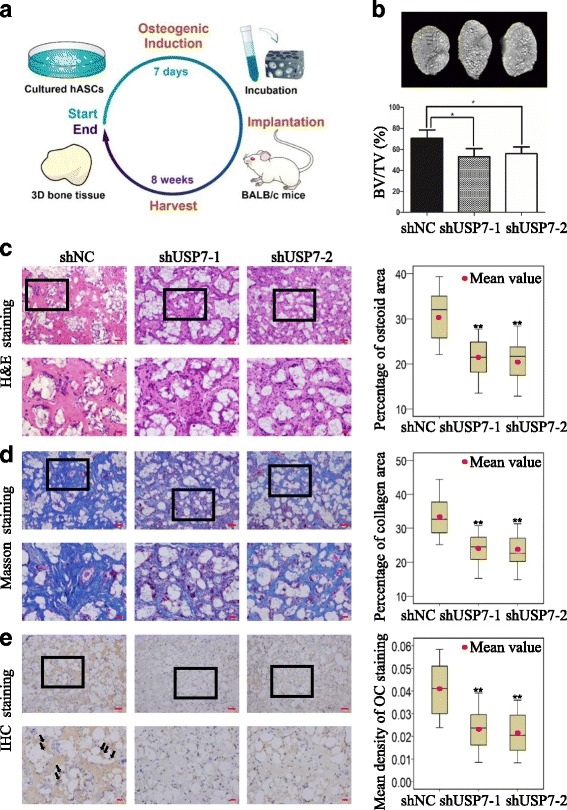
Knockdown of USP7 impairs bone formation in vivo. **a** Schematic diagram illustrating the experimental setup. **b** Representative micro-CT images and quantitative analysis of BV/TV. **c–e** H&E staining (**c**), Masson’s trichrome staining (**d**), and immunohistochemistry staining of OC (**e**) conjugated with histomorphometry analysis of histologic sections from implanted hASC-scaffold hybrids. *Dark-brown* granules indicating positive staining are marked by *black arrows*. Low magnification images are provided in the *upper panels*, *scale bars* = 50 μm, while higher magnification images are in the *lower panels* (**c**–**e**) of the stainings, *scale bars* = 20 μm. Results are presented as the mean ± SD, *n* = 3. *P < 0.05, **P < 0.01. *BV/TV* bone volume to total volume, *hASC* human adipose-derived stem cell, *H&E* hematoxylin and eosin, *IHC* immunohistochemical, *NC* negative control, *OC* osteocalcin, *USP7* ubiquitin specific protease 7

To verify our in vitro findings, we next investigated the role of USP7 in bone formation in a xenograft model. hASCs expressing USP7 shRNAs and control shRNA were seeded in beta-tricalcium phosphate scaffolds, and implanted into the subcutaneous tissue of nude mice (Fig. 6a). After 8 weeks, the implantation hybrids were harvested and analyzed. According to micro-CT analysis, USP7 knockdown groups exhibited less new bone formation and more scaffold remnants (Fig. 6b). Quantifications of micro-CT images further displayed that the percentage of bone volume to total volume (BV/TV) in the USP7 knockdown groups was less than the control group (Fig. 6b). Furthermore, histological examination corroborated the findings from the micro-CT analysis. The USP7 deficiency resulted in lower amounts of uniform, acidophilic osteoid tissue as shown by H&E staining (Fig. 6c), and simultaneously lower amounts of organized extracellular matrix with collagen fiber accumulation (blue color as indicated by Masson’s trichrome staining; Fig. 6d). Histomorphometry analysis of bone-like tissues demonstrated that the area of bone formation was markedly decreased in USP7 knockdown groups compared with the control group (Fig. 6c and d). Consistently, immunohistochemical staining and quantitative measurement for OC also displayed that both the range and intensity of the stained granules in osteoblasts were generally decreased in USP7 knockdown groups (Fig. 6e). Taken together, these results supported that USP7 is essential for the osteogenic differentiation of hASCs in vivo.

### Knockdown of USP7 or USP7 inhibitor HBX 41,108 application inhibits adipogenic differentiation of hASCs in vitro

To explore the potential role of USP7 in regulating adipogenic differentiation, hASCs stably expressing USP7 shRNAs and control shRNA were cultured in adipogenic media (AM) for 14 days. According to the results (Additional file 3: Figure S3A), the lipid droplets, manifested by Oil red O staining and quantification, were markedly reduced in USP7-deficient cells compared with control cells. In addition, the depletion of USP7 significantly downregulated the mRNA expression of the adipogenesis-associated genes *PPARγ* and *C/EBPα* (Additional file 3: Figure S3B). Consistently, the treatment of USP7 inhibitor HBX 41,108 also suppressed the adipogenesis of hASCs in a dose-dependent manner, as shown in Additional file 3: Figure S3C and D.

## Discussion

In this study, we demonstrated that inhibition of protein deubiquitinase USP7 suppressed osteogenic differentiation of hASCs in a catalytic activity-dependent manner, while overexpression of USP7 enhanced hASC osteogenesis. Given the potential differences of MSCs from varied sources [32], we further investigated the effects of USP7 on osteogenic differentiation of hBMMSCs and obtained similar evidence. Our findings uncovered the functional and biological roles of USP7 in osteogenic commitment of MSCs, and shed light on the pursuit of USP7 as a novel potential target for stem cell-mediated regenerative medicine.

It has become increasingly clear that protein ubiquitination is important in various cellular processes, including stem cell pluripotency and differentiation, adult stem cell signaling, cellular reprogramming, spermatogenesis, and oogenesis [33, 34]. In order to effectively screen the osteogenesis-related factors, we generated a luciferase reporter system that could be used to quantitatively and rapidly examine the osteogenic differentiation potential of hASCs. Taking advantage of this system, we performed a preliminary screening with siRNAs targeting deubiquitinases and revealed that USP7 is an essential regulator for osteogenesis. More importantly, genome-wide screening with this reporter system will provide more useful information on the molecular mechanisms of osteogenic differentiation.

As a protein deubiquitinase that cleaves ubiquitin linkages from its substrates, USP7 functions to oppose ubiquitinations of various proteins including E3 ligases (MDM2/MDMX [35] and ICP0 [36]), chromatin associated factors (DNMT1 [37], UHRF1 [38], PHF8 [39], and Tip60 [40]), as well as tumor suppressors (p53 [41], PTEN [42], and claspin [43]). Among these targets, histone demethylase PHF8 is a critical epigenetic factor in cell fate determinations of stem cells [44]. Recently, Han’s study indicated that PHF8 triggered osteogenic differentiation of BMMSCs and facilitated bone formation and regeneration via epigenetically modulating the activity of a nuclear matrix protein, special AT-rich sequence-binding protein 2 (SATB2) [45]. Moreover, it has been reported that USP7 is physically associated with PHF8 and functionally promotes stabilization of PHF8 [39]. Collating these findings, we speculate that USP7-promoted osteogenic differentiation of hASCs could be dependent on USP7-regulated PHF8 stabilization. Since USP7 acts to remove ubiquitination of histone H2B120 lysine (H2BK120Ub) and usually associates with gene transcription repression complex [46, 47], it is unlikely that USP7 promotes osteogenic differentiation through directly regulating transcription of osteogenic-associated genes. We believe that quantitative measurement of the human ubiquitin-modified proteome (ubiquitinome) in USP7-deficient hASCs under osteogenic induction will be helpful in revealing USP7 targeting proteins and understanding the molecular mechanism of USP7-promoted osteogenic differentiation.

MSCs are pluripotent progenitors with multilineage differentiation potentials, capable of undergoing osteogenesis, adipogenesis, and chondrogenesis. Although a balance exists between osteogenesis and adipogenesis of hASCs [48], our study revealed that USP7 depletion also suppressed adipogenesis of hASCs in vitro, indicating that USP7 serves as a positive regulator of MSC differentiation, but not a switcher or balancer of different lineage commitment. Similar observations were reported in a recent research of angiopoietin-like protein 2 (Angptl2) [49], which declared that Angptl2 siRNA inhibited both osteoblast and adipocyte differentiation of ST2 cells under each promoting condition. However, the molecular mechanism of how USP7 promotes and orchestrates different lineage commitment of hASCs remains to be investigated.

In terms of clinical application, since the approval of the first-in-class proteasome inhibitor bortezomib (Velcade®) by the Food and Drug Administration (FDA) for the treatment of relapsed multiple myeloma in 2003 [50], an increasing number of research groups as well as industrial companies have developed more flexible and efficient chemical synthesis protocols to identify better compounds targeting USP7 with high affinity, specificity, cell permeability, and stability. However, despite wide investigation of USP7 inhibitors in tumor therapy [51], little has been explored with regard to their application in the bone engineering field. In our study, HBX 41,108, a cyanoindenopyrazine-derived compound that acts as a potent, reversible, and substrate competitive USP7 inhibitor [30], was used to examine the influence of USP7 deubiquitinase activity on osteogenic differentiation. We demonstrated that this bioactive inhibitor of USP7 significantly facilitated repression of osteogenic genes, and consequently inhibited osteogenic differentiation of hASCs. Moreover, cell viability and apoptosis assays revealed that HBX 41,108 at concentrations of less than or equal to 1 μM had negligible influence on cell proliferation and apoptosis, which excluded the possibility of cell number discrepancy. Thereby, USP7 inhibitors may serve as a potential therapeutic for hyperplasia of bone formation. Since HBX 41,108 has been reported as a potential anticancer drug [52], our findings present the point that application of USP7 inhibitors may impair osteogenic ability and cause related side effects such as osteoporosis during antineoplastic therapy. However, definitive evidence of the correlation between the usage of USP7 inhibitors and bone homeostasis or bone development, to our knowledge, has not yet been reported, and this awaits our further investigation. Therefore, our findings not only broaden the insight of USP7 functionality, but also provide a new and valuable method in the bone tissue engineering field; in particular, prevention or buffering of excessive bone formation.

## Conclusions

In summary, our results demonstrated that protein deubiquitinase USP7 functioned as an essential factor in osteogenic differentiation of hASCs through its catalytic activity. Moreover, we obtained similar results on the effects of USP7 on osteogenic differentiation of hBMMSCs, which indicated the essential biological roles of USP7 in mesenchymal stem cells. Besides unraveling the function of USP7 in osteogenic differentiation, our observations also contribute to the understanding of molecular mechanisms governing osteogenic differentiation of hASCs. To some extent, we provide valuable information on underlying targets to develop highly specific agents for the field of bone tissue engineering.

## Additional files

Additional file 1: Figure S1. Western blotting analysis of USP7 expression in hASCs stably expressing FLAG tagged USP7/wild-type (WT) with antibodies against the indicated proteins. (PDF 12 kb)

Additional file 2: Figure S2. The effects of HBX 41,108 on the apoptosis and proliferation of hASCs. (A) hASCs were treated with proliferation or osteogenic media in the presence of vehicle or HBX 41,108. Apoptosis was evaluated by AnnexinV-FITC apoptosis detection kit. Dot plots are representative of two similar experiments. Positive control: cell suspension boiled at 60 °C for 5 min. (B) Growth curves of hASCs cultured in different concentrations of HBX 41,108. Results are presented as the mean ± SD, *n* = 3. *P < 0.05, **P < 0.01. *hASC* human adipose-derived stem cell, *OM* osteogenic media, *PM* proliferation media. (PDF 127 kb)

Additional file 3: Figure S3. Knockdown of USP7 or HBX 41,108 inhibits adipogenic differentiation of hASCs in vitro. (A) Images of Oil red O staining in shNC, shUSP7-1, and shUSP7-2 groups on day 14 of adipogenic induction, *scale bars* = 100 μm. Histograms show quantification of Oil red O staining by spectrophotometry. (B) Relative mRNA expression of *PPARγ* and *C/EBPα* measured by qRT-PCR in shNC, shUSP7-1, and shUSP7-2 groups on day 14 of adipogenic induction. (C) Images of Oil red O staining in the presence of vehicle or HBX 41,108 on day 14 of adipogenic induction, *scale bars* = 100 μm. Histograms show quantification of Oil red O staining by spectrophotometry. (D) Relative mRNA expression of *PPARγ* and *C/EBPα* measured by qRT-PCR in the presence of vehicle or HBX 41,108 on day 14 of adipogenic induction. Results are presented as the mean ± SD, *n* = 3. *P < 0.05, **P < 0.01. *AM* adipogenic media, *hASC* human adipose-derived stem cell, *PM* proliferation media. (PDF 239 kb)

## Abbreviations

ALP: Alkaline phosphatase
AM: Adipogenic media
AZR: Alizarin red S
BV/TV: Bone volume to total volume
C/EBPα: CCAAT/enhancer binding protein alpha
DMEM: Dulbecco’s modified Eagle’s medium
DUB: Deubiquitinase
GFP: Green fluorescent protein
hASC: Human adipose-derived stem cell
HAUSP: Herpes virus-associated ubiquitin-specific protease
hBMMSC: Human bone marrow-derived mesenchymal stem cell
LSD1: Lysine-specific demethylase 1
Luc: Luciferase
MSC: Mesenchymal stem cell
NC: Negative control
OC: Osteocalcin
OM: Osteogenic media
OSX: Osterix
PBS: Phosphate-buffered saline
PM: Proliferation media
PPARγ: Peroxisome proliferator activated receptor γ
qRT-PCR: Quantitative reverse-transcription polymerase chain reaction
RBP2: Retinoblastoma binding protein 2
REST: Repressor element 1-silencing transcription factor
RT: Room temperature
RUNX2: Runt-related transcription factor 2
SATB2: Special AT-rich sequence-binding protein 2
USP7: Ubiquitin specific protease 7
