# Supplementary figures and images for: Protein deubiquitinase USP7 is required for osteogenic differentiation of human adipose-derived stem cells
Source: Stem Cell Res Ther. 2017 Aug 14;8:186. doi: 10.1186/s13287-017-0637-8 (PMC5557518; doi:10.1186/s13287-017-0637-8)

**Figure S1**

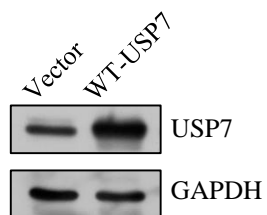

Supplement: Supplementary file 1 — Western blotting analysis of USP7 expression in hASCs stably expressing FLAG tagged USP7/wild-type (WT) with antibodies against the indicated proteins. (PDF 12 kb) [file 13287_2017_637_MOESM1_ESM.pdf]

Figure S2

A

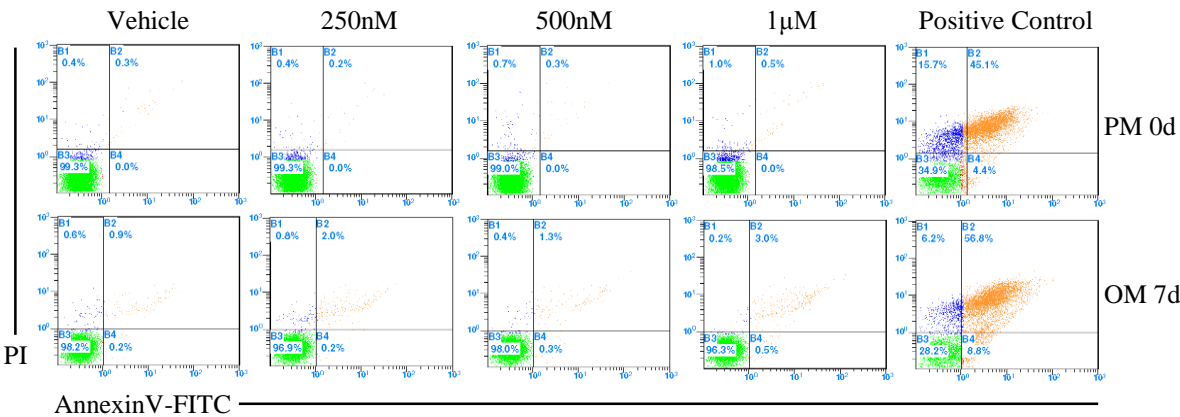

B

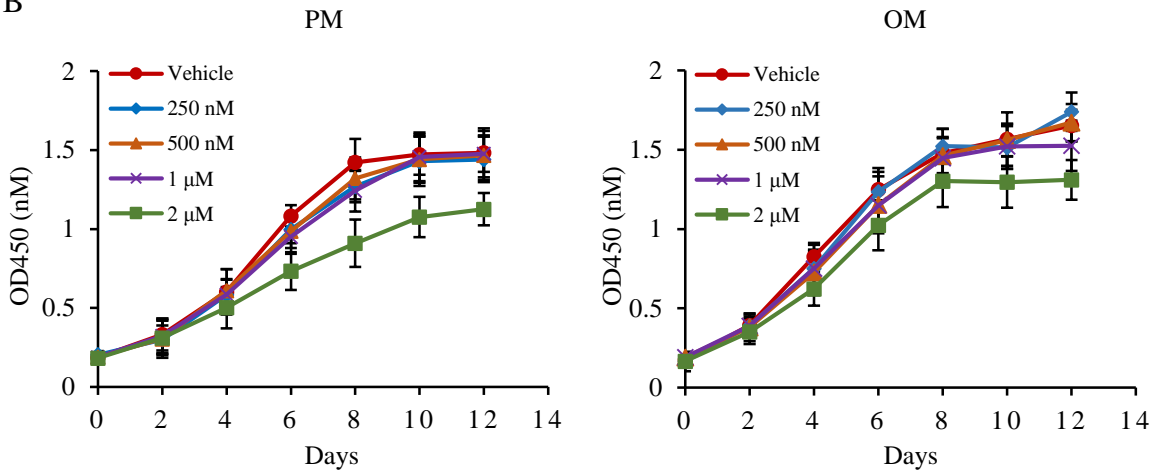

Supplement: Supplementary file 2 — The effects of HBX 41,108 on the apoptosis and proliferation of hASCs. (A) hASCs were treated with proliferation or osteogenic media in the presence of vehicle or HBX 41,108. Apoptosis was evaluated by AnnexinV-FITC apoptosis detection kit. Dot plots are representative of two similar experiments. Positive control: cell suspension boiled at 60 °C for 5 min. (B) Growth curves of hASCs cultured in different concentrations of HBX 41,108. Results are presented as the mean ± SD, n = 3. *P < 0.05, **P < 0.01. hASC human adipose-derived stem cell, OM osteogenic media, PM proliferation media. (PDF 127 kb) [file 13287_2017_637_MOESM2_ESM.pdf]

**Figure S3**

**A**

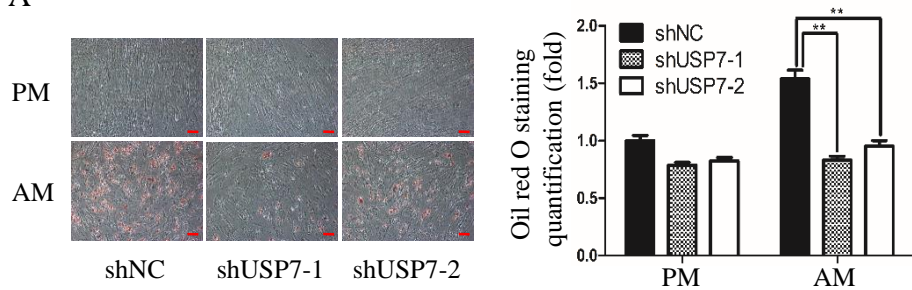

**B**

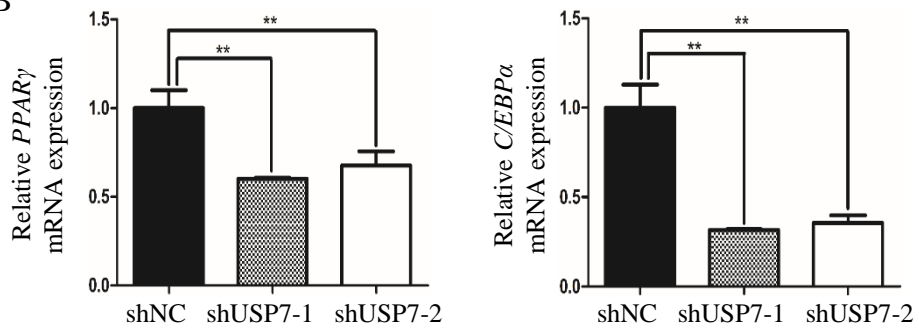

**C**

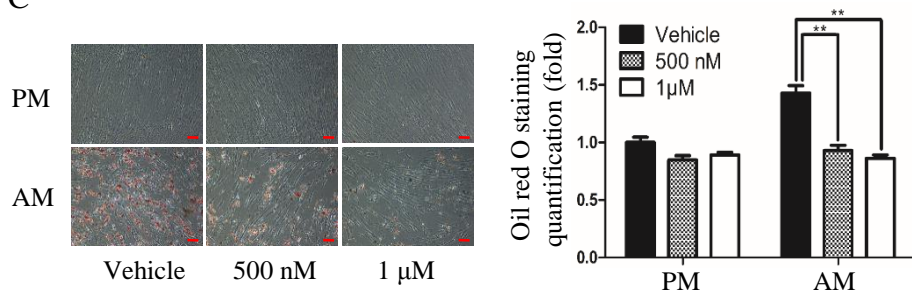

**D**

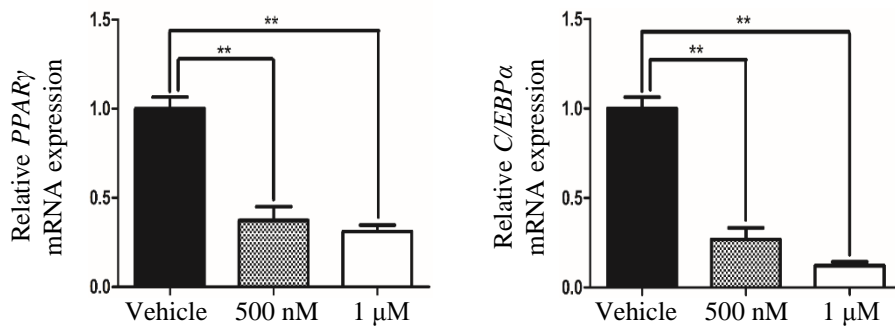

Supplement: Supplementary file 3 — Knockdown of USP7 or HBX 41,108 inhibits adipogenic differentiation of hASCs in vitro. (A) Images of Oil red O staining in shNC, shUSP7-1, and shUSP7-2 groups on day 14 of adipogenic induction, scale bars = 100 μm. Histograms show quantification of Oil red O staining by spectrophotometry. (B) Relative mRNA expression of PPARγ and C/EBPα measured by qRT-PCR in shNC, shUSP7-1, and shUSP7-2 groups on day 14 of adipogenic induction. (C) Images of Oil red O staining in the presence of vehicle or HBX 41,108 on day 14 of adipogenic induction, scale bars = 100 μm. Histograms show quantification of Oil red O staining by spectrophotometry. (D) Relative mRNA expression of PPARγ and C/EBPα measured by qRT-PCR in the presence of vehicle or HBX 41,108 on day 14 of adipogenic induction. Results are presented as the mean ± SD, n = 3. *P < 0.05, **P < 0.01. AM adipogenic media, hASC human adipose-derived stem cell, PM proliferation media. (PDF 239 kb) [file 13287_2017_637_MOESM3_ESM.pdf]
